# CIAPIN1 promotes proliferation and migration of PDGF‐BB‐activated airway smooth muscle cells via the PI3K/AKT and JAK2/STAT3 signaling pathways

**DOI:** 10.14814/phy2.70360

**Published:** 2025-05-07

**Authors:** Ling Zhu, Jin Zhou, Yunfan Gu, Yongtian Xu, Yanfang Guo

**Affiliations:** ^1^ Department of Pediatrics Shanghai Pudong New District Gongli Hospital Shanghai China

**Keywords:** asthma, CIAPIN1, JAK2/STAT3, migration, PI3K/AKT, proliferation

## Abstract

Cytokine‐induced apoptosis inhibitor 1 (CIAPIN1) is an essential anti‐apoptotic protein; however, its role and associated molecular pathways in asthma remain largely unexplored. This study aimed to investigate the potential effects of CIAPIN1 on the proliferation and migration of platelet‐derived growth factor BB (PDGF‐BB)‐induced ASMCs and the underlying mechanisms involved. Considering these aspects, ASMCs are activated with PDGF‐BB as a cellular model for asthma. CIAPIN1 is then downregulated using small interfering ribonucleic acid (siRNA). Western blot analysis was performed to assess protein expression. Elevated levels of CIAPIN1 were observed, demonstrating a positive correlation with cytokine levels. CIAPIN1 expression is significantly increased in PDGF‐BB‐induced human ASMCs. In addition, CIAPIN1 knockdown inhibited proliferation, inflammatory cytokine production, and migration ability, while elevating apoptosis in PDGF‐BB‐induced human ASMCs. Moreover, CIAPIN1 knockdown inhibited phosphorylated phosphoinositide 3‐kinase (p‐PI3K), phosphorylated protein kinase B (p‐Akt), phosphorylated Janus kinase 2 (p‐JAK2), and phosphorylated signal transducer and activator of transcription 3 (p‐STAT3) protein expression. In conclusion, the results indicate that CIAPIN1 regulates the proliferation and migration of human ASMC in response to PDGF‐BB by inhibiting the PI3K/AKT and JAK2/STAT3 pathways.

## 1 INTRODUCTION

Asthma is primarily caused by an allergic response that is closely related to the differentiation of T lymphocytes and cytokine production (Fahy, 2015; Singhania et al., 2018). The causes of asthma are complex, and airway remodeling is a vital aspect of the disease (Banno et al., 2020). In this context, the abnormal proliferation and migration of airway smooth muscle cells (ASMCs) play crucial roles in airway remodeling (Huang et al., 2021). Notably, research has shown that platelet‐derived growth factor‐BB (PDGF‐BB) plays a significant role in stimulating both the proliferation and migration of ASMCs, which are often used as cellular models to study airway remodeling (Huang et al., 2021). This discovery has sparked significant interest in this field.

Cytokine‐induced apoptosis inhibitor 1 (CIAPIN1) is a recently discovered anti‐apoptotic protein expressed extensively in most tissues and localized in the nuclear and cytoplasmic compartments (Hao et al., 2006). The primary role of CIAPIN1 is to impede apoptosis mediated by cytokine deprivation (Kanakura, 2005). When cytokines bind to their respective receptors, they facilitate the initial activation of receptor tyrosine kinases (RTKs) and propagate mitogenic and anti‐apoptotic signals through downstream signaling pathways. Several cytokines, including interleukin 3 (IL‐3), erythropoietin, stem cell factor (CSF), and thrombopoietin, promote the expression of CIAPIN1 via the RTK‐Ras signaling pathway and transcriptional regulation within the IL‐3‐dependent murine pro‐B cell line Ba/F3, thereby demonstrating that CIAPIN1 expression is contingent upon stimulation and signaling from cytokines or growth factors (Kanakura, 2005; Shibayama et al., 2004).

It has been reported that CIAPIN1 may serve as a viable therapeutic target in oncological treatment (Li et al., 2010) through its influence on the ATP binding cassette subfamily B member 1 (ABCB1) (Zhang et al., 2011), thereby enhancing the efficacy of pharmacological agents (Wang et al., 2016), as well as modulating cellular growth and proliferation (Huang et al., 2017) and facilitating cell metastasis (Wang et al., 2019). However, the implications of PDGF‐BB on the proliferation and migration of ASMC remain contentious.

The Janus kinase 2/signal transducer and activator of transcription 3 (JAK2/STAT3) and phosphoinositide 3‐kinase/Akt (PI3K/Akt) signaling pathways are integral components of cellular signal transduction. These pathways are pivotal in numerous physiological and pathological phenomena, including inflammation, stress responses, apoptosis, cell cycle regulation, and cellular proliferation (Wymann et al., 2003; Yu & Jove, 2004). Several scholars have indicated that the activation of Akt downstream of PI3K may mitigate ischemia–reperfusion (I/R) injury (Förster et al., 2006). Furthermore, suppression of the JAK2/STAT3 signaling cascade diminishes apoptosis in intestinal cells subjected to I/R (Wen et al., 2012), renal interstitial fibrosis (Huang et al., 2005), and hypertrophic responses in cardiomyocytes (Abe et al., 2007). However, the impact of CIAPIN1 on the proliferation and migration of PDGF‐BB‐induced ASMCs via the PI3K/AKT and JAK2/STAT3 signaling pathways has not been elucidated. Hence, this study aimed to explore the potential effects of CIAPIN1 on the proliferation and migration of PDGF‐BB‐induced ASMCs and the underlying mechanisms.

## 2 METHODS

### 2.1 Study population

We conducted a cross‐sectional study on patients with asthma admitted to our hospital's Department of Pediatrics' from January 2021 to December 2023. This study included 30 children with acute bronchial asthma (asthma group) and 30 healthy children who underwent physical examination (control group). Asthma was diagnosed based on the 2020 Asthma Guideline Update From the National Asthma Education and Prevention Program (Kaur & Chupp, 2019). The inclusion criteria were as follows: (1) meeting the diagnostic criteria of bronchial asthma; (2) normal cognitive function and cooperation with the study; (3) age <12 years; and (4) complete clinical data. The exclusion criteria were as follows: (1) patients with any malignant hematological diseases or tumors and (2) patients receiving systemic corticosteroids, immunosuppressants, or immunomodulators within 8 weeks. The Ethics Committee of the hospital approved the study protocol. All participants fully understood this research and signed informed consent forms before participating in this study. Fasting peripheral venous blood (5 mL) was collected from all the participants and coagulated at room temperature for 1 h. The sample was centrifuged at 2500 ***g*** and 4°C for 15 min, and the supernatant was divided into 0.5 mL aliquots and stored at −80°C until measurement.

### 2.2 Cell culture

Human ASMCs were purchased from ScienCell Research Labs (Carlsbad, CA, USA) and cultured in high‐glucose DMEM (cat no. 11965084, Gibco, Waltham, MA, USA) and 10% FBS (cat no. 26140079, Gibco, Waltham, MA, USA), supplemented with 100 μg/mL streptomycin and 100 U/mL of penicillin (cat no. 15140122, Gibco, Waltham, MA, USA), ensuring the use of high‐quality reagents. The cells were maintained at 37°C and 5% CO_2_. The medium was changed every 3 days. ASMCs were divided into (1) the control group, which underwent conventional culture and provided crucial baseline data for the experiment, (2) model group (PDGF‐BB: 20 ng/mL) PDGF‐BB (cat no. PHG0041, Gibco, Waltham, MA, USA) treatment for 24 h, (3) silencing control group (PDGF‐BB + siNC), where cells were transfected with siNC for 48 h and stimulated with 20 ng/mL PDGF‐BB for 24 h, and (4) CIAPIN1 silencing group (PDGF‐BB + siCIAPIN1), where cells were transfected with siCIAPIN1 for 48 h and stimulated with 20 ng/mL PDGF‐BB for 24 h.

### 2.3 RNA interference

To knock down CIAPIN1 expression in ASMCs, the cells were transfected with 25 nM CIAPIN1 siRNA (siCIAPIN1) or control scramble siRNA (siNC). The siRNA sequences targeting human CIAPIN1 are listed in Table S1. We transfected ASMCs using Lipofectamine 3000 reagent (cat no. L3000001, Invitrogen, Carlsbad, CA, USA) according to the manufacturer's instructions. Knockdown of CIAPIN1 was confirmed by RT‐qPCR and Western blotting 48 h after transfection.

### 2.4 Cell viability

ASMCs (1 × 10^4^ cells per well) were seeded into 96‐well plates (cat no. 12–565‐135, Thermo Fisher Scientific, Waltham, USA) and treated with saline, PDGF‐BB, PDGF‐BB + siNC, and PDGF‐BB + siCIAPIN1 for 24 h. After this treatment, 10 μL of CCK‐8 solution (cat no. C0037, Beyotime, China) was added to each well. Following a 3‐h incubation, a microplate reader (cat no. A51119500C, Thermo Fisher Scientific, Waltham, MA, USA) was used to measure the cell absorbance at 450 nm.

### 2.5 5‐ethynyl‐20‐deoxyuridine (EdU) assay

ASMC cells were seeded in 96‐well plates at a density of 5 × 10^4^ cells per well for the 5‐ethynyl‐2′‐deoxyuridine (EdU) (cat no. A10044, Thermo Fisher Scientific, Waltham, MA, USA) assay. The cells were subsequently incubated with EdU solution at a concentration of 50 μM and then fixed with 4% paraformaldehyde (cat no. orb90556, Thermo Scientific, Waltham, USA). Following fixation, the nuclei were stained with DAPI dye (cat no. D21490, Thermo Fisher Scientific, Waltham, USA). Images of the cells were captured using an Olympus microscope (CX23LEDRF (S1/S2), Olympus, Japan) (plate_number_1) and analyzed using ImageJ software from the National Institutes of Health, Bethesda, MD, USA.

### 2.6 Cell apoptosis

Apoptotic cells were measured using an Annexin V‐FITC assay kit (cat no. CA1020, Solarbio, Beijing, China). Cells were seeded in a 24‐well plate (5 × 10^4^ cells/well) and treated for 24 h. After trypsin digestion, the cells were treated for 15 min at room temperature with Annexin FITC and propidium iodide (PI). The rate of cell apoptosis was measured by flow cytometry.

### 2.7 Enzyme‐linked immunosorbent assay (ELISA)

Serum was collected from 30 asthma patients and 30 healthy controls. CIAPIN1 (cat no. P9446, Fine Test, Wuhan, China), TGF‐β1 (cat no. DY240, R&D Systems, Minneapolis, USA), MMP‐2 (cat no. MMP200, R&D Systems, Minneapolis, USA), and MMP‐9 (cat no. DMP900, R&D Systems, Minneapolis, USA) levels were measured in the serum. In addition, the culture media of the ASMCs were collected. The secretion levels of TNF‐α (cat no. DTA00D, R&D Systems, Minneapolis, USA), IL‐1β (cat no. DLB50, R&D Systems, Minneapolis, USA) and IL‐6 (cat no. D6050B, R&D Systems, Minneapolis, USA) were measured using ELISA kits (R&D Systems, Minneapolis, USA).

### 2.8 Cell migration assay

The Transwell test was used to examine the migratory capabilities of ASMC. Transwell chambers (8 μm, 1 × 10^5^ cells/mL) were filled with 100 μL of DMEM medium (without FBS). The bottom chamber contained 600 μL DMEM with 10% FBS. After a 72‐h incubation period, the cells in the upper chamber were gently removed using a cotton swab. To visualize the migrating cells, 0.1% crystal violet was used. The average number of migrated cells was determined by counting the labeled cells from five fields under a light microscope (415500–0051‐000, CARL ZEISS, Germany) (magnification: ×200).

### 2.9 Quantitative reverse transcription polymerase chain reaction (RT‐qPCR)

TRIzol (cat no. 15596018, Invitrogen, USA) was used to isolate total RNA from the ASMC. Reverse transcription was then used to generate complementary DNA (cDNA) from total RNA. To amplify the mRNA, Real‐Time Quantitative PCR (RT‐qPCR) was performed using SYBR Green reagent (cat no. 1RR420, TaKaRa, Japan) on an ABI Prism 7700 Real‐Time PCR apparatus (Applied Biosystems, USA). The internal control gene GAPDH was used to normalize relative gene expression using the $2^{-\Delta \Delta C_{t}}$ method. Primers for human CIAPIN1, TNF‐α, IL‐1β, IL‐6, MMP‐2, MMP‐9, and GAPDH were designed using the widely accepted and reliable NCBI Primer‐BLAST Tool, as detailed in Table S2.

### 2.10 Western blotting

Protein specimens were acquired after cellular breakdown using RIPA lysis buffer (cat no. P0013B, Beyotime, China). A BCA kit (cat no. P0012, Beyotime, China) was used to determine protein concentration, followed by the addition of an equal protein volume (40 μg), which was then combined with loading buffer (cat no. P0015, Beyotime, China) and subjected to denaturation in a boiling water bath for 3 min. Upon arrival of bromophenol blue in the separation gel, electrophoresis was commenced at 80 V for 30 min, followed by a subsequent period of 1–2 h at 120 V. Proteins were transferred onto membranes within an ice bath at 300 mA for 60 min. Following a rinsing process of the membranes for 1–2 min using a washing solution, they were either inactivated for 1 h at room temperature or sealed overnight at 4°C. The membranes received treatment with the primary antibodies against CIAPIN1 (1:500, ab154904, rabbit polyclonal, Abcam), p‐PI3K (p85, Tyr458) (1:400, ab278545, rabbit monoclonal, Abcam), PI3K (1:500, ab302958, rabbit monoclonal, Abcam), p‐Akt (Ser473) (1:400, ab81283, rabbit monoclonal, Abcam), Akt (1:400, ab8805, rabbit polyclonal, Abcam), p‐JAK2 (1:200, ab195055, rabbit polyclonal, Abcam), JAK2 (1:200, ab108596, rabbit monoclonal, Abcam), p‐STAT3 (Y705) (1:500, ab267373, rabbit monoclonal, Abcam), STAT3 (1:500, ab68153, rabbit monoclonal, Abcam), and GAPDH (1:2000, ab8245, mouse monoclonal, Abcam) on a shaking table for 1 h at room temperature. The membranes underwent a series of washing steps using a washing solution thrice within a 10 m before and after 1 h of exposure to the secondary antibody at ambient temperature. Subsequently, the membranes were introduced into the developing solution and observed using chemiluminescence imaging analysis equipment (Gel Doc XR, Bio‐Rad).

### 2.11 Statistical analysis

The mean ± standard deviation (SD) of at least three independent experiments was used to express all data. Statistical analyses were performed using SPSS 20.0 (SPSS, Chicago, IL, USA) or GraphPad Prism 9.0 software. The differences between several groups were examined using one‐way ANOVA and post‐hoc Tukey test. Asthma incidence between the various groups was analyzed using Fisher's exact test. Statistical significance was deemed a *p* value of <0.05.

## 3 RESULTS

### 3.1 Serum level and correlation analysis of CIAPIN1 in asthma patients

**FIGURE 1:**
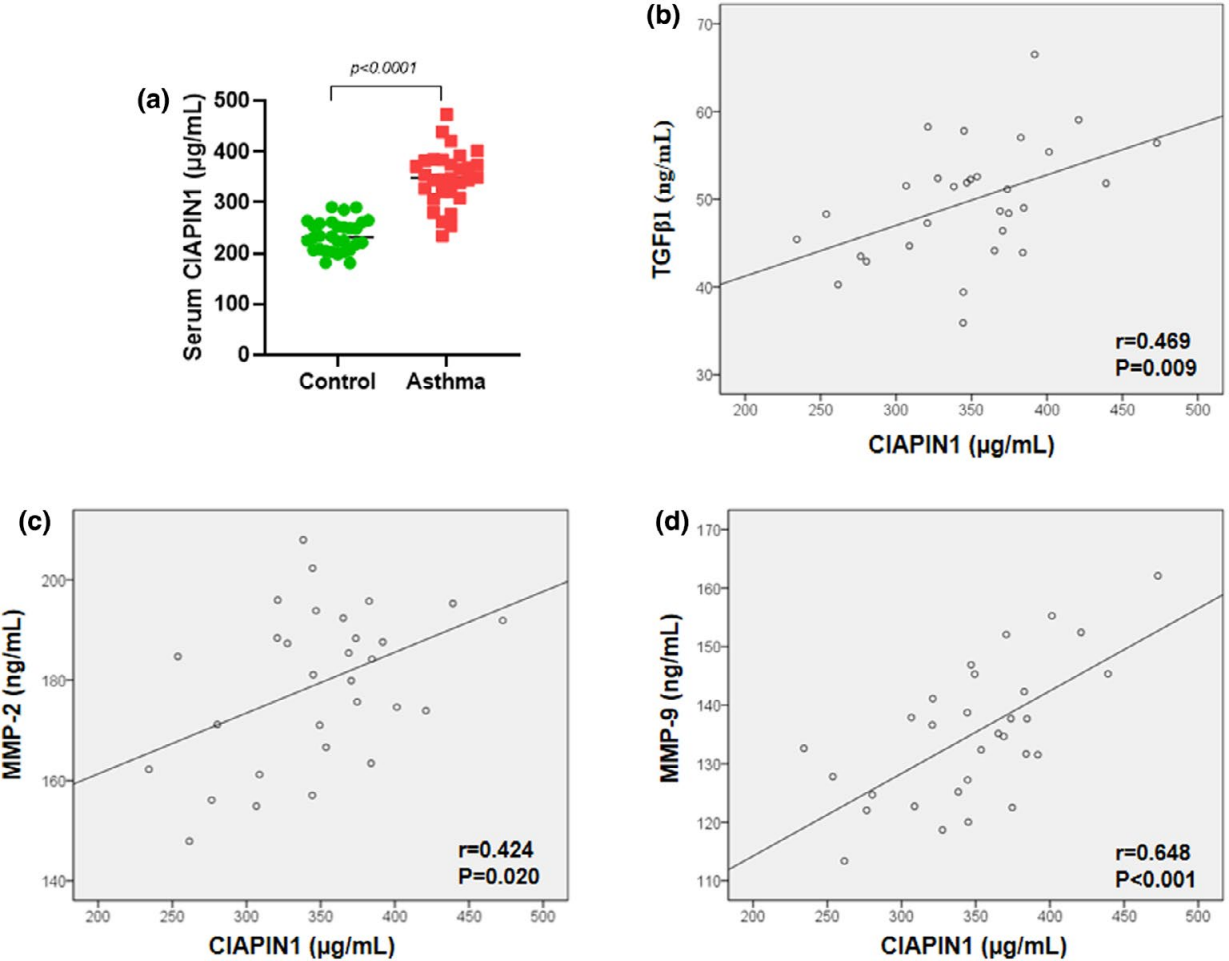
Serum CIAPIN1 is highly expressed in patients with asthma. (a) Serum CIAPIN1 level in healthy subjects and asthma patients was measured by ELISA (*n* = 30 in each group). Serum CIAPIN1 levels are positively correlated with (b) TGF‐β1 (*r* = 0.469, *p* = 0.000), (c) MMP‐2 (*r* = 0.424, *p* = 0.020), and (d) MMP‐9 (*r* = 0.648, *p* < 0.001). Pearson correlation test was performed.

Enzyme‐linked immunosorbent assay (ELISA) analysis revealed that serum levels of CIAPIN1 were significantly higher in patients with asthma than in healthy controls (Figure 1a, *p* < 0.0001). These results suggest that increased CIAPIN1 levels may be associated with the development of asthma.

Pearson's correlation analysis was conducted to examine the relationship between CIAPIN1 expression levels and the clinicopathological characteristics of asthma patients. The results revealed significant positive correlations between CIAPIN1 and TGF‐β1 (*r* = 0.469, *p* = 0.000), MMP‐2 (*r* = 0.424, *p* = 0.020), and MMP‐9 (*r* = 0.648, *p* < 0.001) (Figure 1b–d).

### 3.2 CIAPIN1 highly expressed in PDGF‐BB‐induced human ASMCs

To analyze the expression of CIAPIN1 in human airway smooth muscle cells (ASMCs) induced by PDGF‐BB, the cells were treated with varying concentrations of PDGF‐BB: 0, 1, 5, 10, 20, and 50 ng/mL for 24 h. The CCK‐8 assay was used to assess the viability of ASMCs. The results indicated that ASMC proliferation increased with higher doses of PDGF‐BB (Figure S1a). To evaluate CIAPIN1 mRNA expression, we performed RT‐qPCR on ASMCs treated with different concentrations of PDGF‐BB. The analysis revealed that CIAPIN1 expression increased as the PDGF‐BB dose increased (Figure S1b). Additionally, we conducted western blot analysis of ASMCs treated with PDGF‐BB at the concentrations above, confirming that CIAPIN1 expression also increased with higher doses of PDGF‐BB (Figure S1c). After quantifying the data from the blots, we observed consistent trends in CIAPIN1 expression across ASMCs (Figure S1d). These findings demonstrated that CIAPIN1 expression was significantly increased in PDGF‐BB‐induced ASMCs.

### 3.3 CIAPIN1 knockdown inhibited human ASMCs proliferation

**FIGURE 2:**
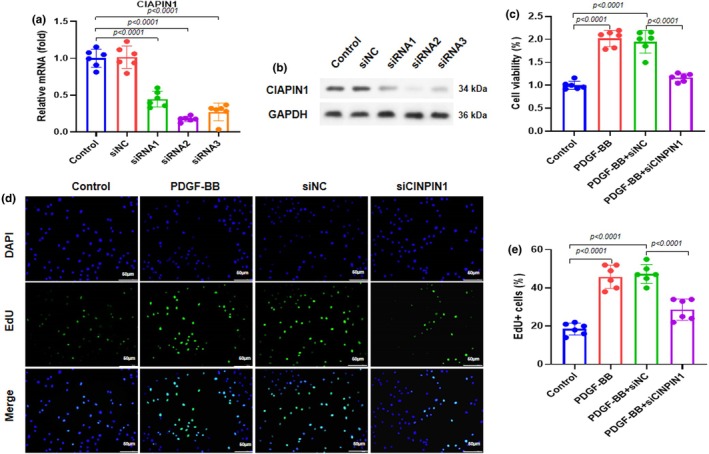
CIAPIN1 knockdown inhibits human ASMC proliferation in the presence of PDGF‐BB. CIAPIN1 siRNA (siCIAPIN1) and control siRNA (siNC) were transfected to ASMCs for 48 h. (a) RT‐qPCR was performed to assess the mRNA expression of CIAPIN1. (b) A western blot was performed to assess the protein expression of CIAPIN1, and representative gel blots of CIAPIN1 were shown. The #2 sequence shows the best inhibitory effect and was chosen for the next experiments. ASMCs were transfected with siCIAPIN1 or siNC and incubated with 20 ng/mL PDGF‐BB for 24 h. (c) Cell viability was assessed by CCK‐8 assay. (d) 5‐ethynyl‐20‐deoxyuridine (EdU) assay was used to determine cell proliferation of ASMCs, and representative images are shown. (e) Quantification of positive cells relative to DAPI‐positive cells. Data are presented as mean ± SD in triplicates and analyzed using one‐way ANOVA, and the Bonferroni test was used for the post‐hoc test.

We transfected ASMCs with CIAPIN1 siRNA (siCIAPIN1) and control siRNA (siNC) for 48 h. Then, we performed RT‐qPCR to assess the mRNA expression of CIAPIN1. The results showed that CIAPIN1 expression was markedly reduced in siRNA1, siRNA2, and siRNA3 compared with the control and siNC (Figure 2a). In addition, we carried out western blot analysis to evaluate the protein expression of CIAPIN1 and observed that siRNA2 (#2 sequence) showed more inhibitory effects (Figure 2b). Sequence #2 was chosen for subsequent experiments. ASMCs were transfected with siCIAPIN1 or siNC, followed by incubation with 20 ng/mL PDGF‐BB for 24 h. Cell viability was examined using the CCK‐8 assay. The results indicated that ASMCs proliferation was increased for PDGF‐BB and PDGF‐BB + siNC, whereas it was remarkably reduced by PDGF‐BB + siCIAPIN1 treatment compared to PDGF‐BB and PDGF‐BB + siNC (Figure 2c). The 5‐ethynyl‐20‐deoxyuridine (EdU) assay was used to determine ASMC proliferation. PDGF‐BB and PDGF‐BB + siNC treatment increased cell proliferation, whereas PDGF‐BB + siCIAPIN1 treatment significantly decreased cell proliferation (Figure 2d). Quantification of EdU‐positive cells relative to DAPI‐positive cells indicated consistent trends of cell proliferation observed by the EdU assay (Figure 2e). These results demonstrated that CIAPIN1 knockdown inhibited human ASMCs proliferation.

### 3.4 CIAPIN1 knockdown elevated human ASMC apoptosis

Cell apoptosis was evaluated by Annexin V‐FITC double staining and analyzed using flow cytometry. The results showed that ASMCs apoptosis was reduced by PDGF‐BB and PDGF‐BB + siNC, whereas it was remarkably elevated by PDGF‐BB + siCIAPIN1 treatment compared with that of PDGF‐BB and PDGF‐BB + siNC (Figure S2a). After quantification analysis, we observed consistent results (Figure S2b). These results demonstrated that CIAPIN1 knockdown increased human ASMC apoptosis.

### 3.5 CIAPIN1 knockdown suppressed PDGF‐BB‐stimulated inflammatory cytokine production in human ASMCs

**FIGURE 3:**
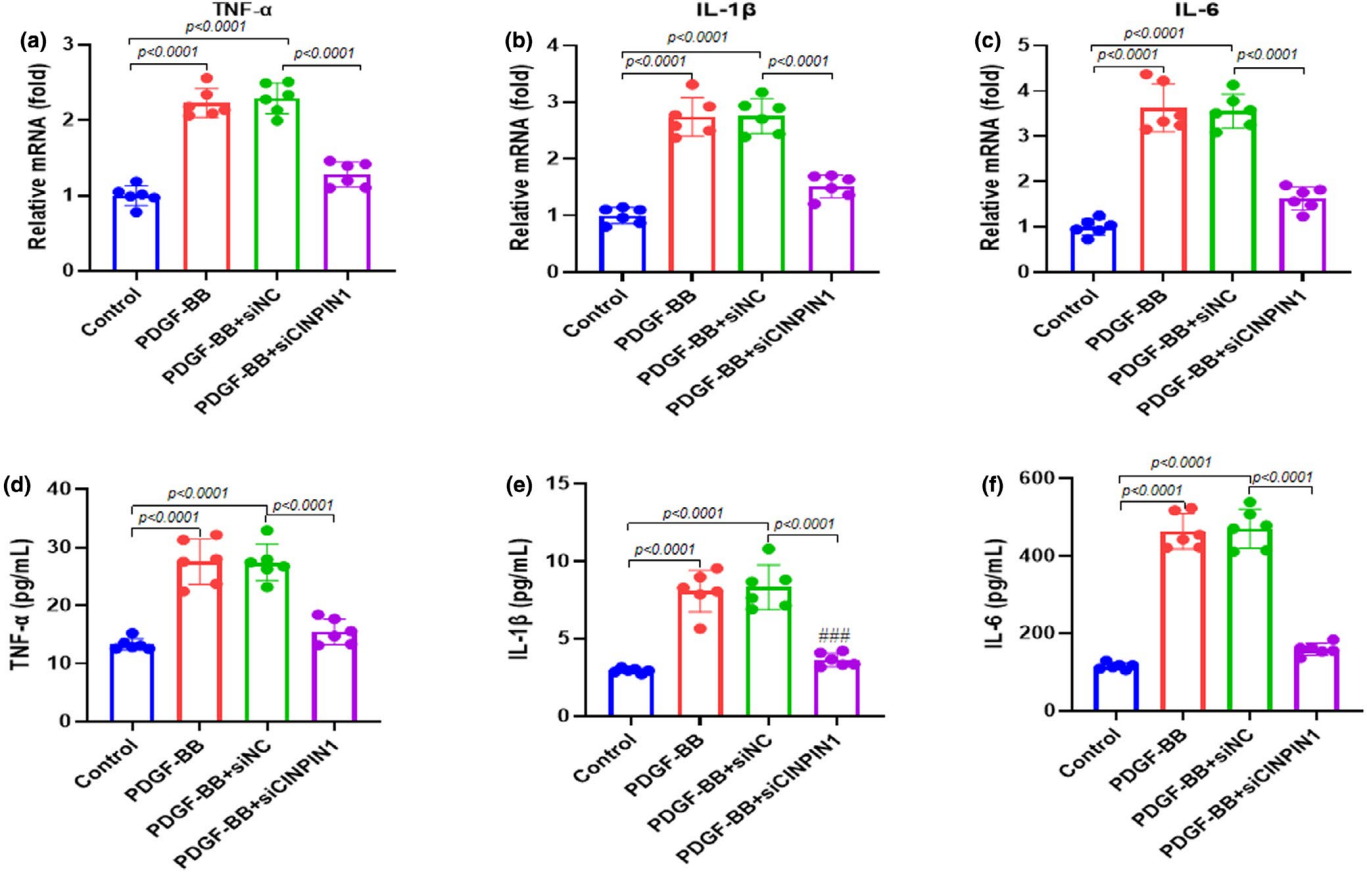
CIAPIN1 knockdown suppresses PDGF‐BB‐stimulated inflammatory cytokines production. RT‐qPCR was used to assess mRNA expression of pro‐inflammatory cytokines (a) TNF‐α, (b) IL‐1β, and (c) IL‐6 in ASMC supernatants stimulated by 20 ng/mL PDGF‐BB. ELISA was used to detect the levels of (d) TNF‐α, (e) IL‐1β, and (f) IL‐6 in ASMCs supernatants stimulated by 20 ng/mL PDGF‐BB. Data are presented as mean ± SD in triplicates and analyzed using one‐way ANOVA and Bonferroni test for the post‐hoc test.

We carried out RT‐qPCR and ELISA to examine the impact of CIAPIN1 knockdown on PDGF‐BB‐stimulated inflammatory effects on ASMCs. RT‐qPCR was used to assess the mRNA expression of pro‐inflammatory cytokines, including TNF‐α, IL‐1β, and IL‐6, in ASMC supernatants stimulated with 20 ng/mL PDGF‐BB. The results showed that PDGF‐BB and PDGF‐BB + siNC treatment elevated pro‐inflammatory cytokines, whereas PDGF‐BB + siCIAPIN1 treatment significantly decreased these pro‐inflammatory cytokines (Figure 3a–c). Moreover, we performed an ELISA assay to evaluate TNF‐α, IL‐1β, and IL‐6 levels in ASMC supernatants stimulated by 20 ng/mL PDGF‐BB. We observed that ASMC inflammatory effects were increased for PDGF‐BB and PDGF‐BB + siNC, whereas they were remarkably reduced by PDGF‐BB + siCIAPIN1 treatment compared to PDGF‐BB and PDGF‐BB + siNC (Figure 3d–f). The results mentioned above showed that CIAPIN1 knockdown attenuated PDGF‐BB‐stimulated inflammatory cytokine production.

### 3.6 CIAPIN1 knockdown attenuated PDGF‐BB‐induced human ASMCs migration

**FIGURE 4:**
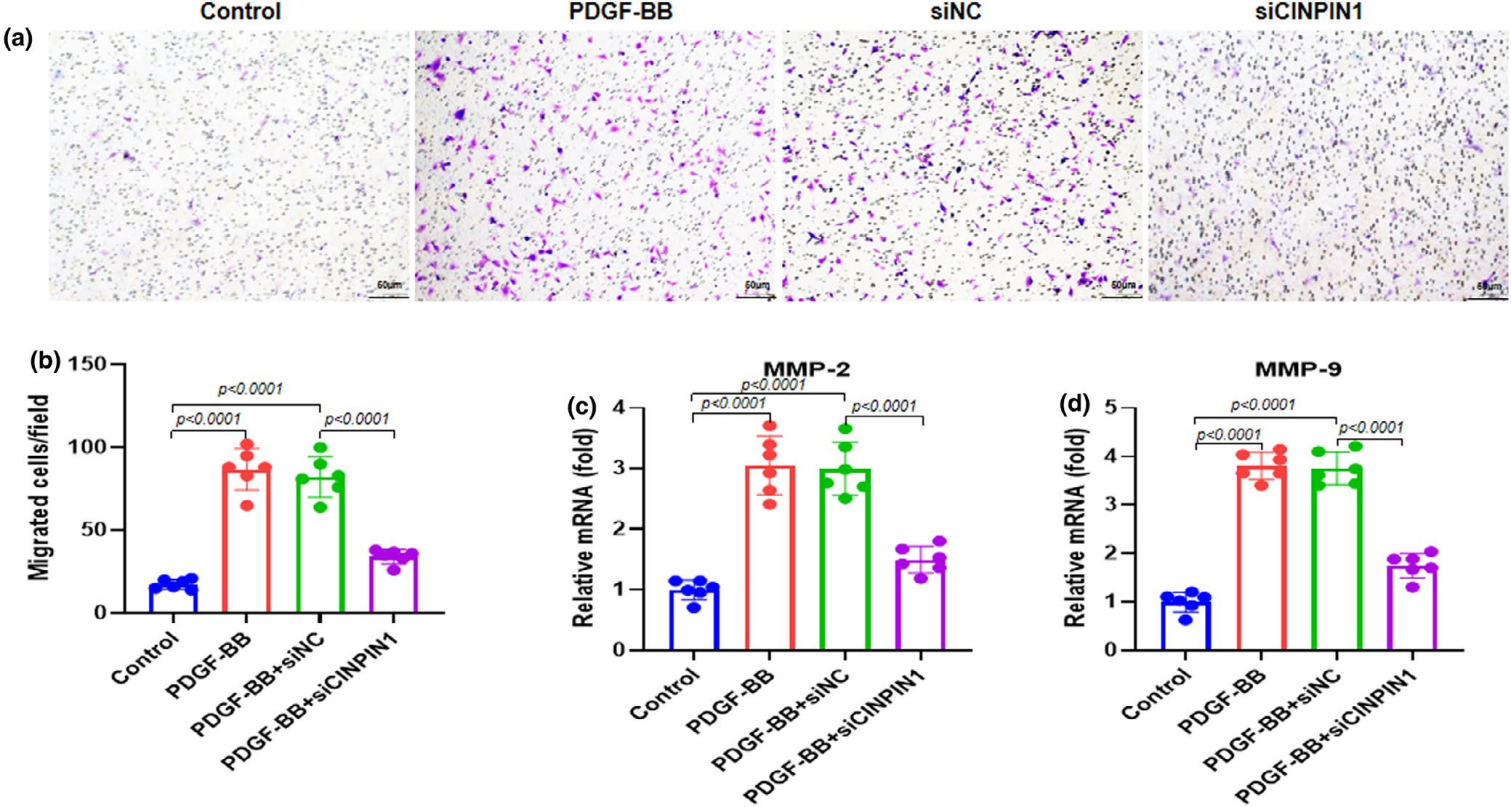
CIAPIN1 knockdown inhibits human ASMC migration in the presence of PDGF‐BB. (a) Transwell assay was used to assess the migration ability of ASMCs with 0.1% crystal violet staining. Representative images of migrated cells are shown (magnification 100×). (b) Quantification of the number of migrated cells. RT‐qPCR was used to assess mRNA expression of genes related to migration (c) MMP‐2 and (d) MMP‐9. Data are presented as mean ± SD in triplicates and analyzed using one‐way ANOVA and Bonferroni test for the post‐hoc test.

We performed a transwell assay and RT‐qPCR to evaluate the effect of CIAPIN1 knockdown on the PDGF‐BB‐induced migration ability of ASMCs. The Transwell assay was used to assess the migration ability of ASMCs with 0.1% crystal violet staining. We observed that PDGF‐BB and PDGF‐BB + siNC treatment elevated migration ability, whereas PDGF‐BB + siCIAPIN1 treatment significantly reduced migration ability (Figure 4a). Quantification analysis showed the same trend for migration ability (Figure 4b). Additionally, RT‐qPCR was used to assess the mRNA expression of genes related to migration, including MMP‐2 and MMP‐9. The results revealed that PDGF‐BB and PDGF‐BB + siNC treatment elevated the mRNA expression of MMP‐2 and MMP‐9, whereas PDGF‐BB + siCIAPIN1 treatment significantly lowered the mRNA expression of MMP‐2 and MMP‐9 (Figure 4c,d). These results indicated that CIAPIN1 knockdown inhibited PDGF‐BB‐induced human ASMC migration.

### 3.7 CIAPIN1 knockdown inhibited the PI3K/Akt and JAK2/STAT3 pathways

**FIGURE 5:**
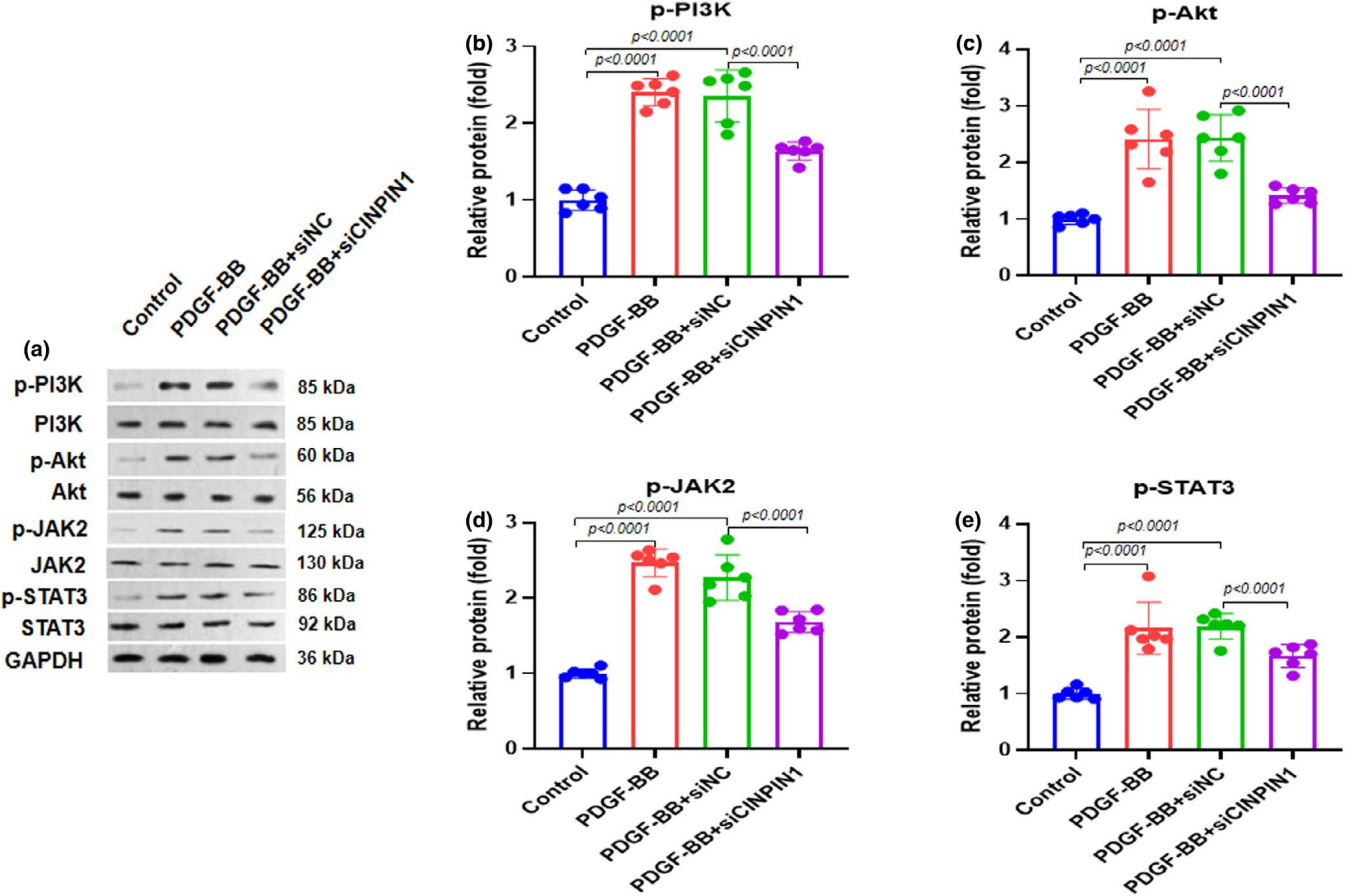
CIAPIN1 knockdown modulates PI3K/Akt and JAK2/STAT3 pathway. (a) Representative gel blots depicting levels of phosphorylated PI3K (p‐PI3K, p85, Tyr458, normalized to total PI3K), phosphorylated Akt (p‐Akt, Ser473, normalized to total Akt), phosphorylated JAK2 (p‐JAK2, normalized to total JAK2), phosphorylated STAT3 (p‐STAT3, normalized to total STAT3). (b–e) Quantification analysis of p‐PI3K, p‐Akt, p‐JAK2, and p‐STAT3 protein bands. Data represent the average of three independent experiments (mean ± SD).

We carried out western blot analysis to investigate the impact of CIAPIN1 knockdown on the PI3K/Akt and JAK2/STAT3 pathways. We observed that PDGF‐BB and PDGF‐BB + siNC treatment elevated the protein expression of p‐PI3K, p‐Akt, p‐JAK2, and p‐STAT3, whereas PDGF‐BB + siCIAPIN1 treatment significantly reduced the protein expression of p‐PI3K, p‐Akt, p‐JAK2, and p‐STAT3 (Figure 5a). Quantification of p‐PI3K, p‐Akt, p‐JAK2, and p‐STAT3 protein bands revealed the same trends in protein expression patterns (Figure 5b–e). These results indicated that CIAPIN1 knockdown inhibited the PI3K/Akt and JAK2/STAT3 pathways.

## 4 DISCUSSION

Asthma, a condition that is commonly triggered by several environmental and cellular factors, poses a significant risk to human health (Moheimani et al., 2016; Pawankar, 2014). The recent escalation of asthma has not only led to a considerable economic burden but also underscores the need for cost‐effective solutions. The condition is increasingly recognized as a complex syndrome with diverse clinical presentations (Sockrider & Fussner, 2020), and its management is complicated by its intricate etiology and pathophysiology (Jones et al., 2018). Despite extensive investigations into its origins and various therapeutic modalities, an optimal treatment regimen is yet to be identified, necessitating further inquiry to elucidate asthma pathogenesis and develop effective therapeutic interventions (Kwah & Peters, 2019).

The present study demonstrated the impact of CIAPIN1 on the proliferation and migration of PDGF‐BB‐induced ASMCs and the underlying mechanisms. Our data revealed that CIAPIN1 levels were elevated and positively associated with asthma severity. CIAPIN1 expression was significantly increased in PDGF‐BB‐induced human ASMCs. In addition, CIAPIN1 knockdown inhibited proliferation, inflammation, and migration, while elevating apoptosis in PDGF‐BB‐induced human ASMCs. Moreover, CIAPIN1 knockdown attenuated the PI3K/Akt and JAK2/STAT3 signaling pathways. Thus, these results suggest that CIAPIN1 could have a significant impact on the proliferation and migration of PDGF‐BB‐induced ASMCs via the PI3K/AKT and JAK2/STAT3 signaling pathways.

Airway remodeling, a significant contributor to asthma development, is a complex interplay that is influenced by the proliferation and migration of smooth muscle cells in the airways (Gao et al., 2021). To understand the impact of increased CIAPIN1 expression on smooth muscle cells, we conducted siRNA experiments to knock down CIAPIN1. We assessed the proliferation and migratory capabilities of human airway smooth muscle cells (ASMCs) after stimulation with PDGF‐BB. Our findings indicate that stimulation with PDGF‐BB significantly enhanced the proliferation and migration of human ASMCs while concurrently reducing the rate of apoptosis. Conversely, the knockdown of CIAPIN1 diminishes the proliferative and migratory capacities of the cells while increasing apoptosis. These results suggest that the upregulation of CIAPIN1 substantially augments the proliferation and migratory capabilities of human ASMCs and decreases apoptosis in response to PDGF‐BB.

The Janus kinase 2/signal transducer and activator of transcription 3 (JAK2/STAT3) signaling cascade, along with the phosphoinositide 3‐kinase/Akt (PI3K/Akt) signaling pathway, is an essential component of cellular signal transduction. These pathways are crucial for various physiological and pathological processes, including inflammation, stress responses, apoptosis, cell cycle regulation, and cellular proliferation (Wymann et al., 2003; Yu & Jove, 2004). Research has indicated that activation of Akt, which occurs downstream of PI3K, may alleviate the harmful effects of ischemia–reperfusion (I/R) injury (Förster et al., 2006). Furthermore, inhibition of the JAK2/STAT3 signaling cascade has been shown to reduce apoptosis in intestinal cells subjected to I/R (Wen et al., 2012), in cases of renal interstitial fibrosis (Huang et al., 2005), and during the hypertrophic response in cardiomyocytes (Abe et al., 2007). However, in the present investigation, we performed western blot analysis to explore the effects of CIAPIN1 knockdown on the PI3K/Akt and JAK2/STAT3 signaling pathways. Our findings revealed that treatment with PDGF‐BB and PDGF‐BB combined with siNC significantly increased p‐PI3K, p‐Akt, p‐JAK2, and p‐STAT3 protein levels. In contrast, treatment with PDGF‐BB and siCIAPIN1 resulted in a marked decrease in the protein expression of p‐PI3K, p‐Akt, p‐JAK2, and p‐STAT3. Subsequent quantitative analysis of the protein bands corresponding to these phosphorylated proteins confirmed the trends observed in their expression patterns. Our results suggest that CIAPIN1 knockdown inhibits the PI3K/Akt and JAK2/STAT3 signaling pathways.

Asthma significantly reduces quality of life and leads to ongoing lung dysfunction throughout an individual's life, especially in children (Haktanir Abul & Phipatanakul, 2019). This study focused on airway remodeling and investigated the key factors involved in the development of asthma, contributing to our understanding of how the condition arises. Although our findings may serve as an essential step toward developing effective asthma treatments, it is crucial to emphasize that more research is urgently needed to translate our cellular models into suitable animal models for in vivo testing, which will inform future clinical applications. CIAPIN1 plays a role in the PI3K/AKT and JAK2/STAT3 signaling pathways, making it a promising candidate for therapeutic interventions to manage asthma.

While this study provides valuable insights, it is crucial to acknowledge its limitations. First, PDGF‐BB was employed to activate ASMCs; however, other growth factors (e.g., TGF‐β, EGF, and FGF) and inflammatory cytokines (for example, IL‐4, IL‐13, and TNF‐α) may also contribute to ASMC dysfunction in diseases such as asthma. Second, although the PI3K/AKT and JAK2/STAT3 pathways were examined, other pathways (e.g., MAPK, NF‐κB, and Wnt/β‐catenin) may also be implicated in CIAPIN1‐mediated effects. Third, pharmacological inhibitors (for example, LY294002 for PI3K and AG490 for JAK2) may exhibit off‐target effects, potentially affecting the data interpretation. Finally, the study results were not validated using in vivo models.

## 5 CONCLUSION

**FIGURE 6:**
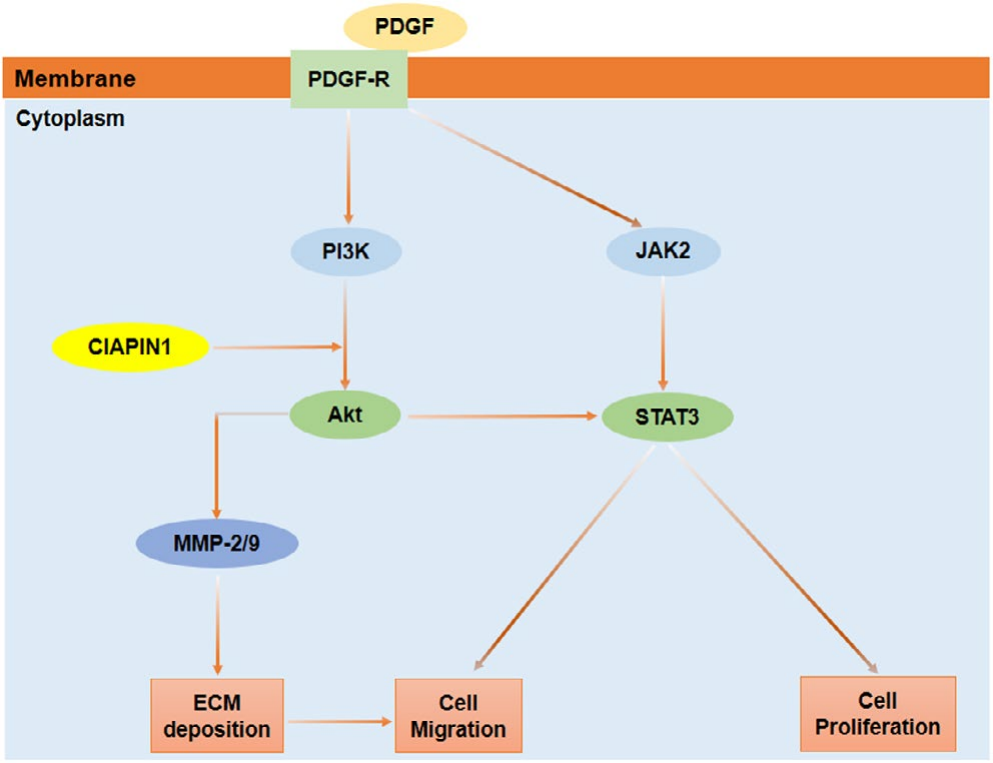
Schematic diagram of effect of CIAPIN1 on proliferation and migration of ASMCs.

In conclusion, this study demonstrated that CIAPIN1 regulates the proliferation and migration of human ASMCs in response to PDGF‐BB by inhibiting the PI3K/AKT and JAK2/STAT3 signaling pathways (Figure 6). Additionally, reducing CIAPIN1 expression may be a promising therapeutic strategy for alleviating asthma.

## AUTHOR CONTRIBUTIONS

Ling Zhu: Conceptualization, data curation, investigation, methodology, writing—original draft preparation. Jin Zhou, Yunfan Gu, Yongtian Xu: Data curation, investigation, methodology, formal analysis, writing—review and editing. Yanfang Guo: Writing—review and editing, supervision, project administration, methodology, investigation, funding acquisition, formal analysis, data curation.

## FUNDING INFORMATION

This study was supported by the Important Weak Disciplines in Pudong New Area, Shanghai (PWZbr2022‐06).

## CONFLICT OF INTEREST STATEMENT

The authors report no conflicts of interest in this work.

## ETHICS STATEMENT

The Ethics Committee of Shanghai Pudong New District Gongli Hospital approved (GLYY1s2024‐052) this study. The authors followed all standard protocols in accordance with the 1964 Declaration of Helsinki. All subjects provided informed consent to participate in the study.

## Supporting information


Figure S1.

Table S1.

Appendix S1.

## Data Availability

Due to confidentiality issues, the datasets generated and/or analyzed during the current work are not publicly available but are available from the corresponding author upon reasonable request.
