# Supplementary material for: CIAPIN1 promotes proliferation and migration of PDGF‐BB‐activated airway smooth muscle cells via the PI3K/AKT and JAK2/STAT3 signaling pathways
Source: Physiol Rep. 2025 May 7;13(9):e70360. doi: 10.14814/phy2.70360 (PMC12058325; doi:10.14814/phy2.70360)
Supplement: Supplementary file 1 — Figure S1. [file PHY2-13-e70360-s003.pdf]

CIAPIN1 promotes proliferation and migration of PDGF-BB-activated airway smooth muscle cells via the PI3K/AKT and JAK2/STAT3 signaling pathways

Ling Zhu, Jin Zhou, Yunfan Gu, Yongtian Xu, Yanfang Guo✉

Supplementary Figures

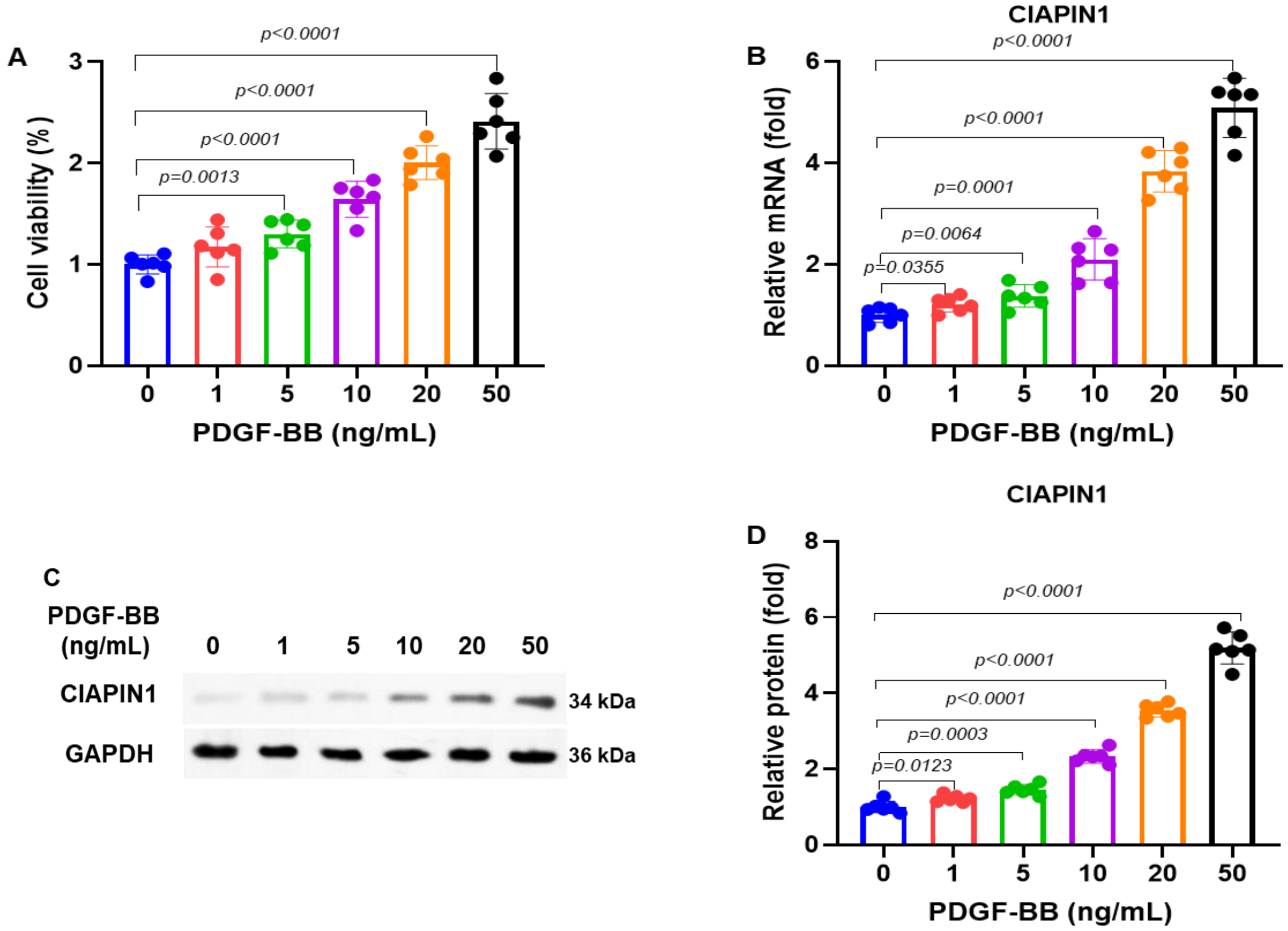

FIGURE S1 CIAPIN1 is highly expressed in PDGF-BB-stimulated human ASMCs. ASMCs were treated with 0, 1, 5, 10, 20, and 50 ng/mL PDGF-BB for 24 h. (A) The cell viability of ASMCs was determined by CCK-8 assay. (B) RT-qPCR determined the CIAPIN1 mRNA expression in ASMCs treated with PDGF-BB at different concentrations. (C) Representative gel blots of CIAPIN1 by Western blotting in ASMCs treated with PDGF-BB at different concentrations. (D) These protein blots were quantified by normalizing them to GAPDH. Data are presented as mean ± SD in triplicates and analyzed using one-way ANOVA, and the Dunnett test was used for the post-hoc test.

Supplementary Figures

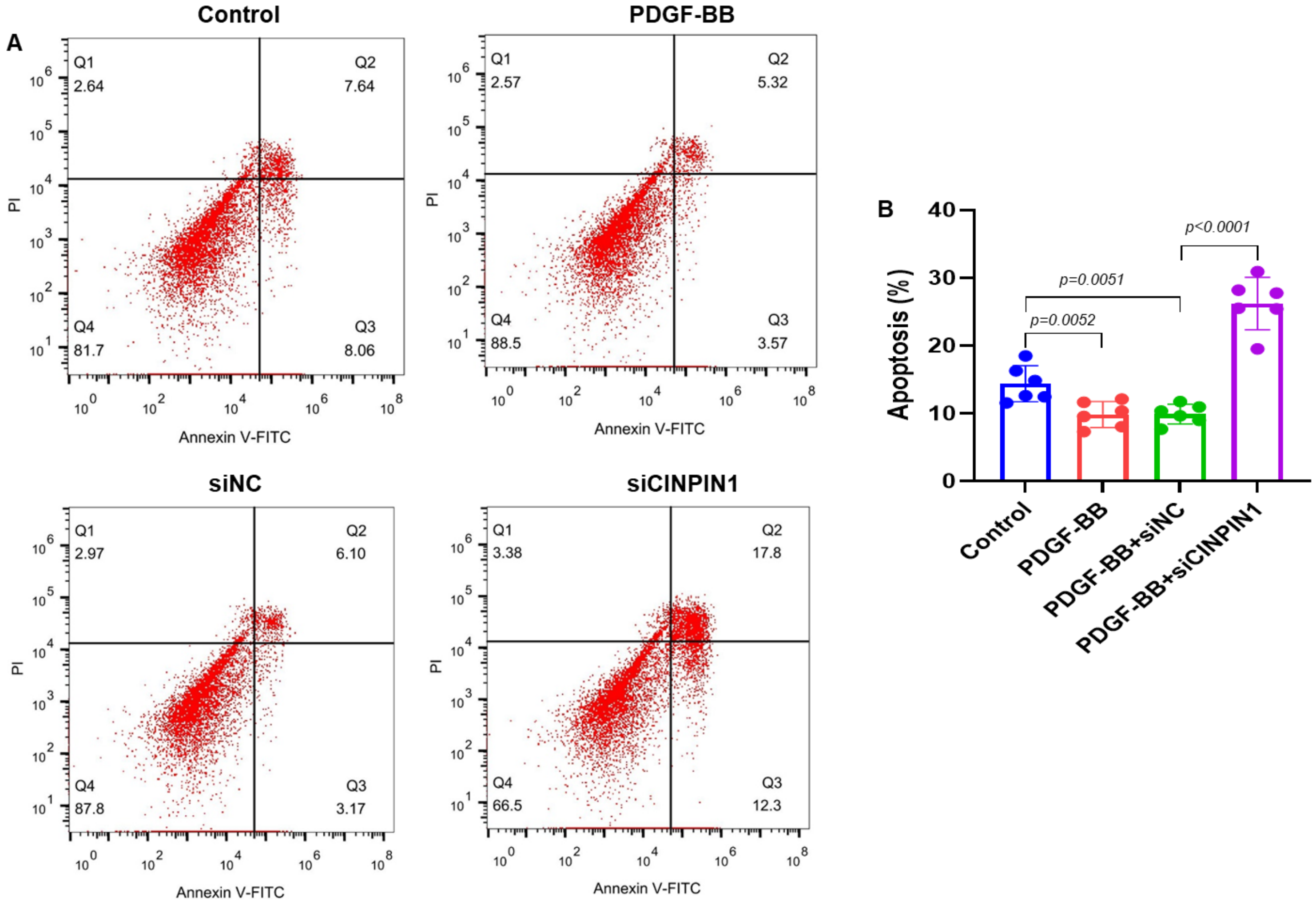

FIGURE S2 CIAPIN1 knockdown enhances human ASMC apoptosis. (A) Cell apoptosis was assessed by Annexin V-FITC double staining and analyzed by flow cytometry. (B) The apoptotic rate was calculated by sum of the lower right quadrant and the upper right quadrant. Data are presented as mean ± SD in triplicates and analyzed using one-way ANOVA, and the Bonferroni test was used for the post-hoc test.
