# Supplementary material for: CIAPIN1 promotes proliferation and migration of PDGF‐BB‐activated airway smooth muscle cells via the PI3K/AKT and JAK2/STAT3 signaling pathways
Source: Physiol Rep. 2025 May 7;13(9):e70360. doi: 10.14814/phy2.70360 (PMC12058325; doi:10.14814/phy2.70360)
Supplement: Supplementary file 2 — Table S1. [file PHY2-13-e70360-s001.docx]

**Table S1** Sequences of CIAPIN1 siRNA used in this study.

| Genes | Forward primer (5′-3′) | Reverse primer (5′-3′) |
| --- | --- | --- |
| Sequences 1 | GGAUAAGCUUCAAGCGUUAACTT | GUUAACGCUUGAAGCUUAUCCTT |
| Sequences 2 | AGACAGCUGUAGAUAACAAUATT | UAUUGUUAUCUACAGCUGUCUTT |
| Sequences 3 | GGUUCUUCUAGGCAGCUUAAGTT | CUUAAGCUGCCUAGAAGAACCTT |

**Table S2** Primer sequences used in this research.

| Genes | Forward primer (5′-3′) | Reverse primer (5′-3′) |
| --- | --- | --- |
| Human CIAPIN1 | CACCAAGAAGTCTTCTCCTTCAGTG | GCTGAGAGGGTCCACAGCT |
| Human TNF-α | CTGGGCAGGTCTACTTTGGG | CTGGAGGCCCCAGTTTGAAT |
| Human IL-1β | CCAGGGACAGGATATGGAGCA | TTCAACACGCAGGACAGGTACAG |
| Human IL-6 | TCCACAAGCGCCTTCGGTC | GGTCAGGGGTGGTTATTGCAT |
| Human MMP-2 | AGCGAGTGGATGCCGCCTTTAA | CATTCCAGGCATCTGCGATGAG |
| Human MMP-9 | GCCACTACTGTGCCTTTGAGTC | CCCTCAGAGAATCGCCAGTACT |
| Human GAPDH | CATGTTGCAACCGGGAAGGA | CGCCCAATACGACCAAATCAG |
