# Supplementary material for: CIAPIN1 promotes proliferation and migration of PDGF‐BB‐activated airway smooth muscle cells via the PI3K/AKT and JAK2/STAT3 signaling pathways
Source: Physiol Rep. 2025 May 7;13(9):e70360. doi: 10.14814/phy2.70360 (PMC12058325; doi:10.14814/phy2.70360)

CIAPIN1 promotes proliferation and migration of PDGF-BB-activated airway smooth muscle cells via the PI3K/AKT and JAK2/STAT3 signaling pathways

Ling Zhu, Jin Zhou, Yunfan Gu, Yongtian Xu, Yanfang Guo✉

Supplementary Files

Original uncropped raw images of western blot panel used in **Figure 2C**

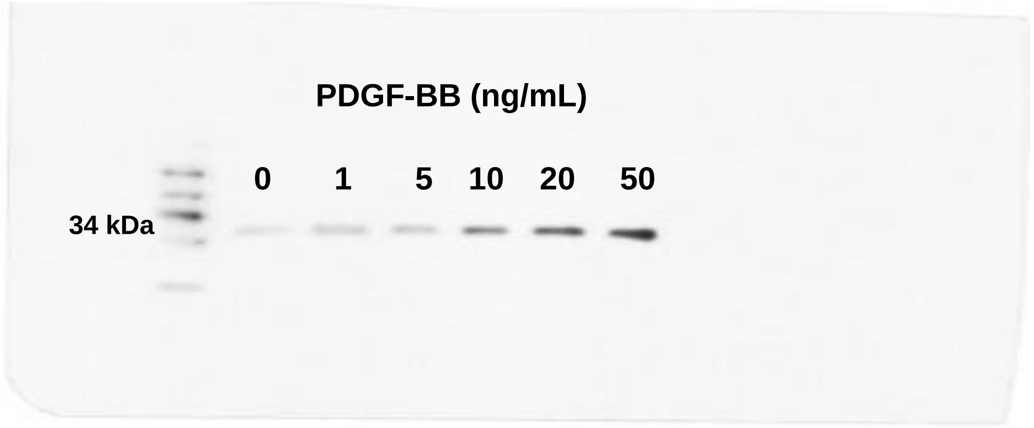

CIAPIN1

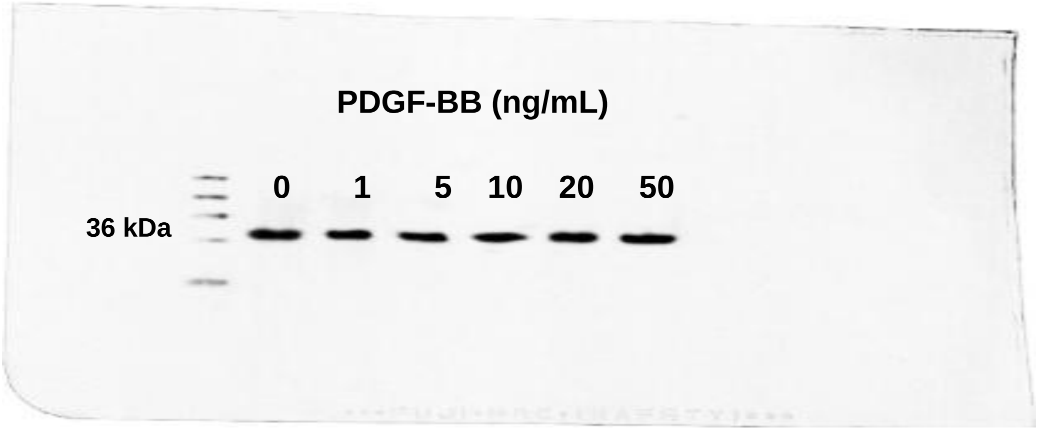

GAPDH

Supplementary Files

Original uncropped raw images of western blot panel used in **Figure 3B**

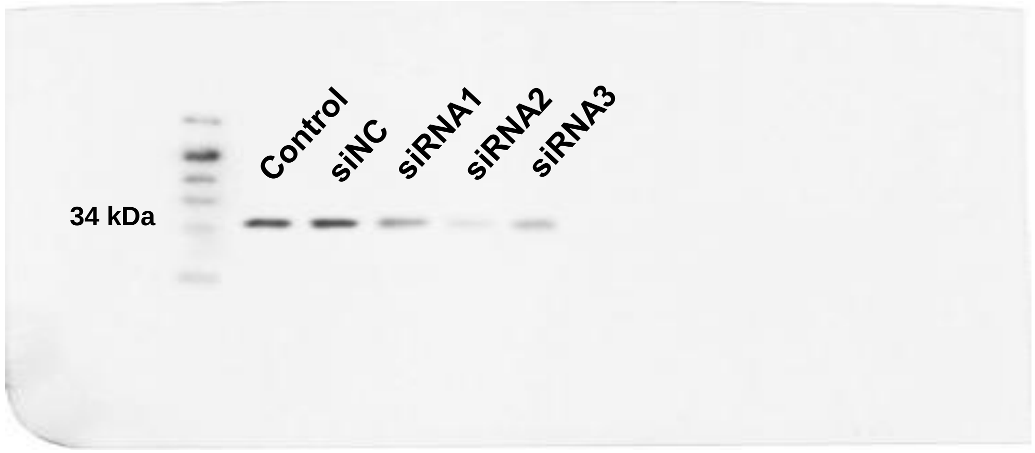

CIAPIN1

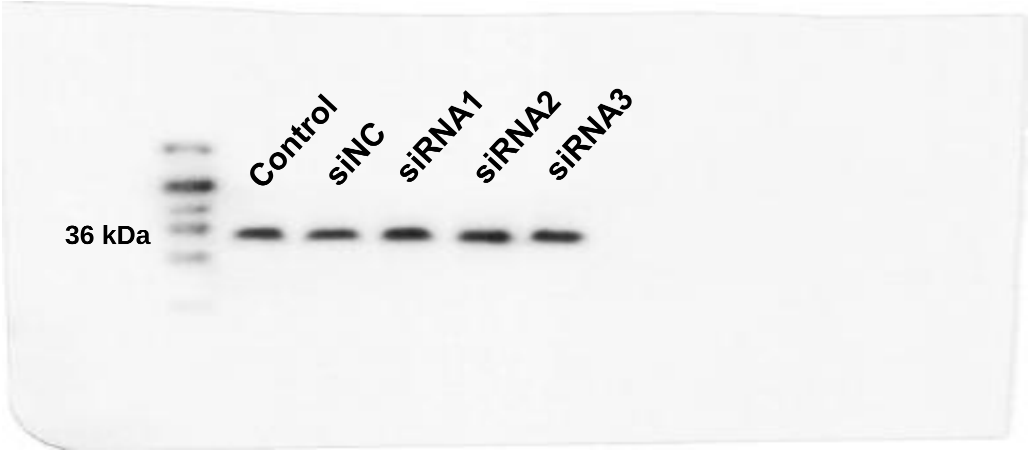

GAPDH

Supplementary Files

Original uncropped raw images of western blot panel used in **Figure 6A**

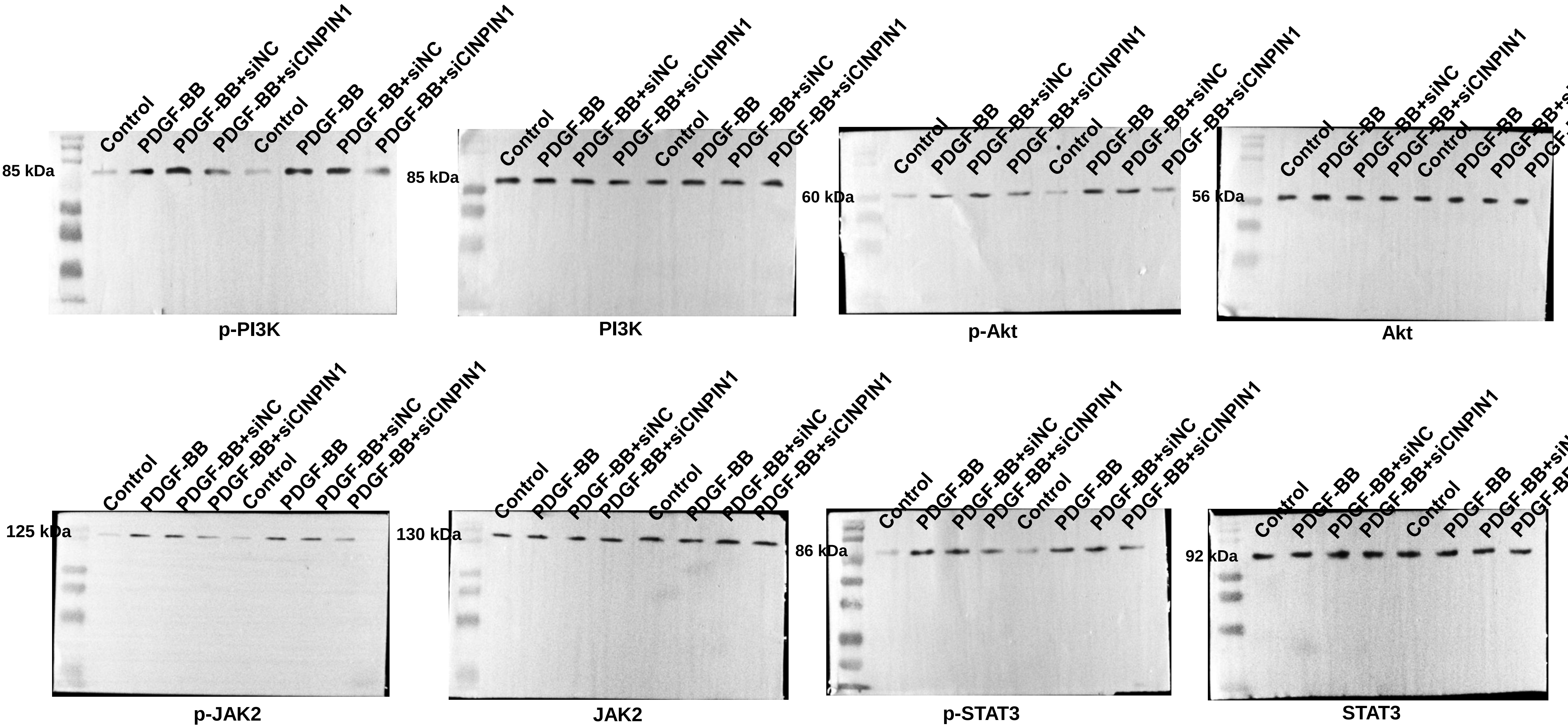

Supplementary Files

Original uncropped raw images of western blot panel used in **Figure 6A**

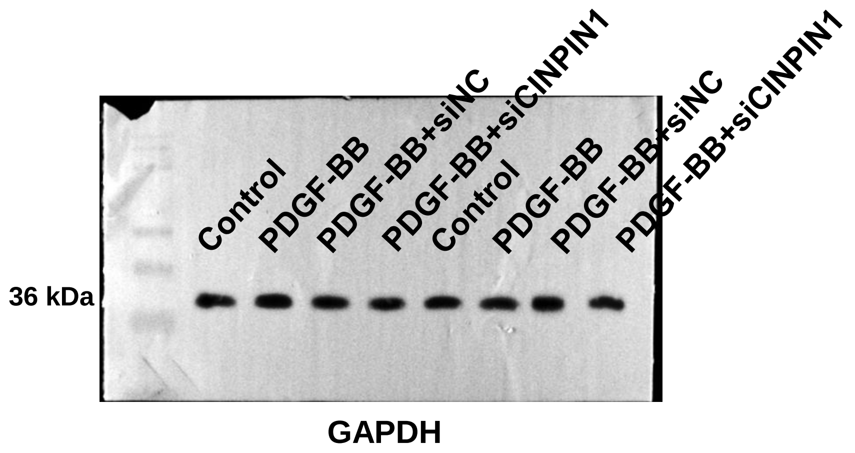

Supplement: Supplementary file 3 — Appendix S1. [file PHY2-13-e70360-s002.pdf]
